# Supplementary material for: Experimental violation of a Bell-like inequality for causal order
Source: Sci Adv. 2026 Jun 10;12(24):eaee2912. doi: 10.1126/sciadv.aee2912 (PMC13251822; doi:10.1126/sciadv.aee2912)
Supplement: Supplementary file 1 — Supplementary Text Figs. S1 to S3 Table S1 [file sciadv.aee2912_sm.pdf]

Science Advances
AAAS

Supplementary Materials for

Experimental violation of a Bell-like inequality for causal order

Yu Guo *et al.*

Corresponding author: Giulio Chiribella, giulio@cs.hku.hk; Bi-Heng Liu, bhliu@ustc.edu.cn

Sci. Adv. **12**, eaee2912 (2026)
DOI: 10.1126/sciadv.aee2912

This PDF file includes:

Supplementary Text
Figs. S1 to S3
Table S1

1 Core experimental innovations

For completeness, we summarize the main experimental innovations of our work in this section.

1. *Quantum switch with polarization control and time-bin target.* Our experiment realizes, for the first time, a photonic quantum switch in which a polarization-encoded control qubit coherently controls operations acting on a time-bin target qubit. Previous optical demonstrations of the quantum switch have typically employed other combinations of degrees of freedom, such as path-controlled polarization, polarization-controlled transverse-electric modes, or path-controlled time-bin qubits. In contrast, our implementation combines polarization control with a time-bin target. This encoding offers a practical advantage: both polarization and path operations can be manipulated rapidly using electro-optic elements, making the architecture particularly well suited to scenarios—such as the present experiment—in which fast operations are required on both the control and target systems. This implementation enriches the experimental toolbox for realizing indefinite causal order in photonic platforms.

2. *Implementation of a measurement-and-repreparation process on a time-bin qubit with delayed readout of the measurement outcomes.* Our experiment implements a time-bin–based strategy to realize the measurement-and-repreparation protocol inside a quantum switch without generating additional spatial modes. By embedding electro-optic (EO) switches in asymmetric Mach–Zehnder interferometers (AMZIs), we coherently manipulate an ancillary time-bin qubit and encode the measurement outcomes in the temporal domain. The outcomes are mapped onto distinct photon arrival times through additional fiber delays, while the repreparation settings are recorded via trigger signals synchronized with the EO switch driving signals. This approach enables deterministic readout of the mesurement outcome and repreparation choice combinations directly in the time domain, avoiding the need for multiple copies of the measurement apparatus required in polarization-based implementations.

3. *Phase stabilization of a kilometer-scale Mach–Zehnder interferometer via temperature control.* Our experiment further develops a temperature-controlled stabilization scheme to ensure stable interference in the quantum switch Mach–Zehnder interferometer under fast time-bin operations. Instead of active phase locking—which is difficult to implement with rapidly

switching optical paths—we minimize the length of the unshared optical paths between the two causal orders and stabilize the entire experimental platform using a custom temperature control system. This design suppresses phase drift induced by environmental fluctuations and enables a long-term interference visibility of about 0.98, which is crucial for observing the violation of the DRF inequality.

2 Details on the entangled photon source

The experiment reported in this work was conducted with a photonic polarization entangled source based on a spontaneous parametric down-conversion process on a type-II cut ppKTP crystal in a Sagnac configuration. A 150 mW diode laser centered at 775 nm was reshaped into a pulse array with a acoustic optical modulator (AOM). The pulse width and repetition rate were 600 ns and 50 kHz, respectively. Setting the polarization of the pump laser to be diagonal, we generated pairs of entangled photons centered at 1550 nm in a polarization state $(|H\rangle|V\rangle + |V\rangle|H\rangle)/\sqrt{2}$, which were then separated by a polarizing beam splitter (PBS). The pump laser was blocked with long-pass and narrow-band pass filters. Then, the entangled photon pairs were coupled into single-mode fibers and transformed into $(|H\rangle|H\rangle + |V\rangle|V\rangle)/\sqrt{2}$ with a fiber polarization controller. The idler photon was distributed to Bob while the signal photon was guided into the quantum switch and finally measured by Charlie. When measuring the photon pairs in the diagonal basis, we observed a visibility of more than 0.99, indicating a good quality of our entangled source.

The duty cycle of our pump pulses was 3% and the threshold power of the AOM we used is 200 mW, which limits the pump power of our entanglement source to less than 6 mW. When setting the coincidence window to 0.5 ns, the observed coincidence rate of the photon source was about 60,000 pairs per second, the counting rate of each detector was about 180,000, and thus the coincidence efficiency was 0.33. The coincidence rate was attenuated to 1400 pairs per second after the signal photon passed through the whole apparatus. The drop in collection rate with and without the quantum switch indicates that the switch introduces a loss of more than 16 dB. This loss is primarily due to three factors: the insertion loss of the optical switch (approximately 1.5 dB each), the fiber beam splitters (3 dB each), and the losses associated with fiber-to-free-space coupling (0.5 dB each).

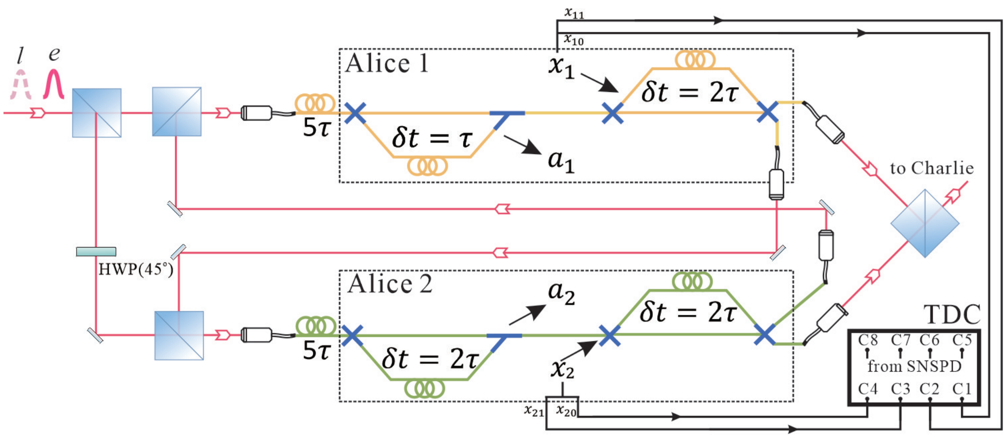

Figure S1: **The reading out of a_i and x_i.** For each Alice, the first asymmetric Mach–Zehnder interferometer (AMZI) is used to perform a measurement on the time-bin target qubit, while the second is used to reset the target state. The time-bin qubit is defined by a relative time delay of $\tau = 1$ μs. To enable the retrieval of the measurement outcomes a_i without disturbing the control qubit, an additional time delay τ is introduced in all AMZIs except for the measurement AMZI of Alice 1. To retrieval the repreparation choices x_i, four trigger signals (amplitude 3.3 V, width 10 ns), synchronized to the resetting signal x_i (the driving signals of OS2 and OS5 in Fig. 2 of the main text), is guided to four channels (C1-C4) of the TDC. The other four TDC channels (C5-C8) are used to record the detected photon events from the SNSPD. TDC, time-to-digital converter; SNSPD, superconducting nanowire single-photon detector.

3 Details on the time-bin encoding and the reading out of a_i and x_j

Time-bin encoding constitutes a robust photonic qubit encoding scheme, wherein the logical basis states are defined by the photon arrival time in two well-separated temporal modes, commonly denoted as the early and late time bins. Such qubits can be generated and coherently manipulated using AMZIs, where the path-length difference between the two arms imposes a fixed temporal delay between the temporal modes. Here the path-length difference is required to be significantly larger than the single photon coherence length to ensure that the early and late bins are well separated in the time domain with negligible temporal overlap. By routing photons through the short or long arm of an AMZI, one can map quantum information onto the temporal degree of freedom while

preserving phase coherence. To measure the time-bin qubit, a second AMZI is typically used to project the temporal superposition onto the target logical basis, followed by post selecting the middle time bin detecting events. Importantly, the measurement AMZI is configured such that the early bin from the preparation stage propagates along the long arm and the late bin along the short arm, which compensates the temporal delay and enables coherent interference.

In our implementation, the time-bin qubit is defined by a relative time delay $\tau = 1$ μs, corresponding to a path length difference of 200 meters between the two fiber arms of the AMZI. The operations on the time-bin qubit are performed at a repetition rate of 0.1 MHz. This rate is primarily limited by the performance of the EO switches. The EO switches used in our experiment have a rising time of less than 100 ns at a switching rate of no more than 0.3 MHz. While its isolation reaches about 30 dB at low switching rates (a few Hz), this isolation degrades as the switching rate increases. At our operating rate of 0.1 MHz, the isolation was measured to be 20-23 dB. Another factor that restricts the operation rate is the overall optical loss of the system. To maintain a sufficiently high counting rate of the photon source, we employed a moderate pump pulse duty cycle. Specifically, we set the pulse width to 600 ns in our experiment. The use of high-performance commercial optical switches (e.g. BATi Nanona fiber switches, rising time < 60 ns, repetition rate up to 1 MHz) could significantly enhance the operation speed of time-bin qubit manipulations, potentially by an order of magnitude.

In our experiment, time-bin encoding plays a central role in implementing the measurement-and-repreparation protocol within the quantum switch, as it allows measurement outcomes a_i and control settings x_i to be recorded in the temporal domain and retrieved at a later stage without disturbing the coherence of the control qubit. To this end, we introduce an ancillary time-bin qubit that defers the readout of measurement results until after the control qubit has been measured.

Specifically, each Alice employs two AMZIs: the first projects the target qubit onto the computational basis to yield the outcome a_i, and the second resets the target qubit in the state $|x_i\rangle$ according to the input x_i. In the measurement AMZI, photons propagating through the long arm correspond to $a_i = 0$, while those passing through the short arm correspond to $a_i = 1$. Conversely, in the repreparation AMZI, routing a photon into the long arm prepares the target qubit in the state $|1\rangle$, whereas the short arm corresponds to preparation in $|0\rangle$. As illustrated in Fig. S1, we introduce an ancillary time-bin qubit by imposing an additional time delay τ in three of the four AMZIs. The

ancillary qubit expands the dimension of the Hilbert space, enabling the measurement outcomes of Alice 1 and Alice 2 to be mapped into sequential distinct temporal modes; the relative delay of the detected signals thus reveals the measurement outcome a_i.

In parallel, we use four trigger pulse signals x_{ij} (($i \in \{1, 2\}, j \in \{0, 1\}$)) to record the repreparation setting choices of Alice 1 and Alice 2. Here, x_{ij} denotes the setting choice j of Alice i and is used to trigger four channels of the time-to-digital converter (TDC, C1-C4 of Fig. S1). The amplitude and width of these x_{ij} signals are set to 3.3 V and 10 ns to satisfy the operating conditions of the TDC. To maintain precise temporal synchronization, the x_{ij} signals are phase-locked to the driving signals of the EO switches. Specifically, x_{10} and x_{11} are synchronized to the low and high levels of the driving signal for OS2, respectively, while x_{20} and x_{21} are synchronized to the low and high levels of the driving signal for OS5. Here, OS2 and OS5 denote the first EO switches in the repreparation AMZIs of Alice 1 and Alice 2, respectively (Fig. 2 of the main text). Under these configurations, all signals a_is and x_is can be deterministically distinguished via the combination of the trigger channel and the relative time delay between the two-photon detection events, as summarized in Tab. S1.

Note that the length of the fibers in the AMZI can be measured with a fluctuation of less than 2 cm. This precision is achieved by comparing the photon arrival times with and without the fiber inserted. Given that the pulse width in our experiment is 600 ns, a 2 cm variation in fiber length is negligible.

4 Timing coordination and spacetime configuration in the experiment

As discussed in the main text, the causal relations among the operations of the four agents are crucial for our experiment. According to Fig. 1(A) of the main text, Alice 1 and Alice 2 lie in the causal past of Charlie, while Bob is spacelike separated from the other three parties. To satisfy these causal relations, both the spatial separations between the agents and the durations of their individual operations must be carefully considered. Except for a 10 cm free-space optical delay line, the four agents are connected via fiber spools. The idler photon is sent to Bob through a 3 kilometer fiber,

Table S1: The time delay and trigger channel settings to read out $a_1x_1a_2x_2$.

$a_1x_1a_2x_2$	relative time delay	trigger detector	$a_1x_1a_2x_2$	relative time delay	trigger detector
0000	3τ	D1D3	1000	2τ	D1D3
0001	5τ	D1D4	1001	4τ	D1D4
0010	τ	D1D3	1010	0	D1D3
0011	3τ	D1D4	1011	2τ	D1D4
0100	5τ	D2D3	1100	4τ	D2D3
0101	7τ	D2D4	1101	6τ	D2D4
0110	3τ	D2D3	1110	2τ	D2D3
0111	5τ	D2D4	1111	4τ	D2D4

corresponding to a delay of 15 μs. Inside the quantum switch, a 1 kilometer fiber is placed before each Alice's laboratory. In addition, the AMZIs have short arms consisting of 1 meter fibers and long arms consisting of 201 meter and 401 meter fibers, respectively. The fiber configuration is shown in Fig. S1, where the unlabeled links are typically 1 meter fibers. In the following discussion, we neglect the delay introduced by these 1 meter fibers, as their propagation delay (about 5 ns) is much smaller than the temporal separation defining our time-bin qubit. Under these conditions, the maximal total length of the path traveled by the signal photon inside the quantum switch is approximately 3.4 kilometers, corresponding to a delay of about 17 μs.

To satisfy these causal relations, a high level of coordination between the optical and electronic subsystems is required. This coordination, as illustrated in Fig. S2, relies on the precise synchronization of all optoelectronic components, including the AOM, random number generators (RNGs), and EO switches. To achieve this, the operating clocks of these devices are synchronized throughout the system. The AOM is driven by a digital signal generator (DSG), while all EO switches are controlled by an FPGA, which provides their driving signals. For two of the EO switches (OS2 and OS5 in Fig. 2 of the main text), these signals are further modulated by outputs from RNGs. The clock output from the DSG (10 MHz) serves as the global synchronization signal for the entire

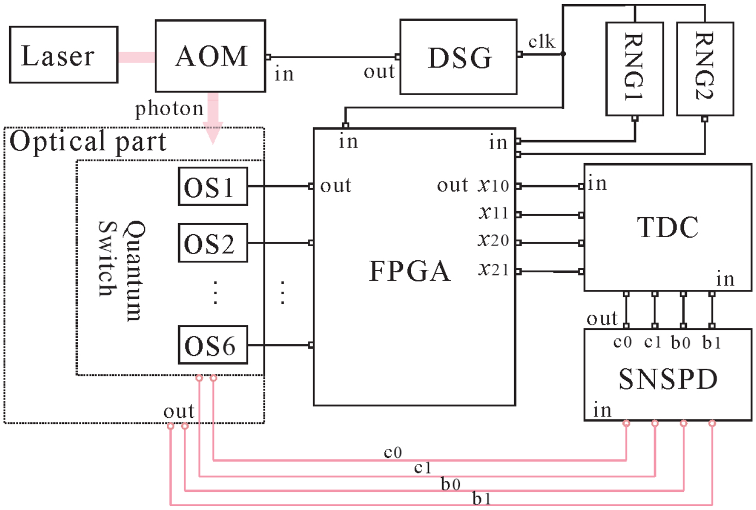

Figure S2: **Electronic control loop of our setup.** The AOM is driven by a DSG, whose clock signal is used to synchronize RNGs, FPGA, and OSs. RNGs are used to randomize the repreparation setting choices of Alice 1 and Alice 2. To read out Alices' measurement outcomes and repreparation setting choices, four pulse signals, labeled as x_{ij} ($i \in \{1, 2\}, j \in \{0, 1\}$), are used to trigger the TDC. These trigger signals are synchronized to Alices' repreparation setting choices. DSG, digital signal generator; AOM, acoustic optical modulator; FPGA, field-programmable gate array; OS, electro-optical switch; RNG, random number generator.

system. The time delay of each electronic control loop can be measured with a precision of a few nanoseconds—well below the time scale defined by the manipulation rate of the time-bin qubit.

Building on this synchronized architecture, we now analyze the detailed timing requirements for photon propagation through the quantum switch and the corresponding operations of the EO switches (OS1-OS6 in Fig. 2 of the main text). Here, OS1 and OS4 are located in the measurement AMZIs of the two Alices, OS2 and OS5 are placed in their repreparation AMZIs, and OS3 and OS6 guide the photons either within the quantum switch or out of it. Each experimental trial lasts 20 μs, during which only a single pair of entangled photons is generated. As discussed above, the generation of the pump pulse, the RNGs, and the measurement-and-repreparation operations performed by Alice 1 and Alice 2 are synchronized by a master oscillator (see Fig. S2). Each trial begins with the generation of a pump pulse at $t = 0$. When the control qubit is in the state $|H\rangle$, the signal photon reaches OS1 at $t = 5$ μs. After passing through the short or long arm of the first AMZI, it reaches OS2 at the earliest at $t = 5$ μs and at the latest at $t = 6$ μs. Subsequently, the

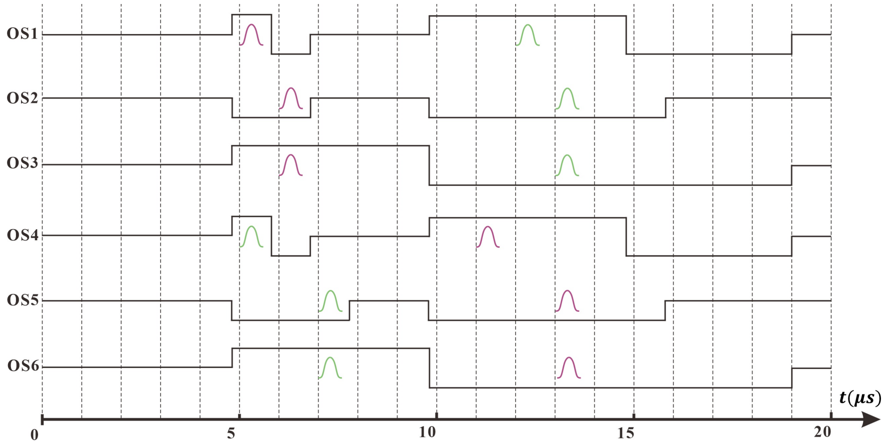

Figure S3: **Typical timing diagram of the driving signals for the EO switches when both Alices measure the initial target qubit $|0\rangle$ in the computational basis and reprepare it in the state $|0\rangle$.** For OS1, OS2, OS4, and OS5, a high-level (3.3 V) driving signal routes the photon to the long arm of the corresponding AMZI, while a low-level (0 V) signal routes it to the short arm. For OS3 and OS6, the high level routes the photon between Alice 1 and Alice 2, whereas the low level routes it out of the quantum switch. All optical switches are initialized at 1.7 V and reset to 1.7 V after $t = 19$ μs.

photon reaches OS3 between $t = 5$ μs and $t = 8$ μs, OS4 between $t = 10$ μs and $t = 13$ μs, OS5 between $t = 10\mu$s and $t = 15$ μs, and OS6 between $t = 10$ μs and $t = 17$ μs. On the other hand, when the control qubit is in the state $|V\rangle$, the signal photon reaches OS4 at $t = 5$ μs. It then reaches OS5 between $t = 5$ μs and $t = 7$ μs, OS6 between $t = 5$ μs and $t = 9$ μs, OS1 between $t = 10$ μs and $t = 14$ μs, OS2 between $t = 10$ μs and $t = 15$ μs, and OS3 between $t = 10$ μs and $t = 17$ μs. The asymmetry between the two cases arises from the different delays in the measurement AMZIs: the relative delay in Alice 2's AMZI is 2 μs, while that in Alice 1's AMZI is 1 μs.

These considerations determine the timing requirements for setting the EO switches. Given that the pulse width is 600 ns, OS1 must be set before $t = 5$ μs and its driving signal must be maintained until $t = 14.6$ μs, while OS4 must be set before $t = 5$ μs and maintained until $t = 13.6$ μs. Similarly, OS2 and OS5 must be set before $t = 5$ μs and their driving signals must be maintained until $t = 15.6$ μs. The switches OS3 and OS6 must be triggered twice during each trial. The first activation routes the photon between Alice 1 and Alice 2 after the photon passes through the switches for the first time, while the second activation guides the photon out of the quantum switch

after the second passage. In our experiment, OS3 is first set before $t = 5\ \mu$s and its driving signal is maintained until $t = 8.6\ \mu$s; it is then set again before $t = 10\ \mu$s and maintained until $t = 15.6\ \mu$s. Similarly, OS6 is first set before $t = 5\ \mu$s and maintained until $t = 9.6\ \mu$s, and is then set again before $t = 10\ \mu$s and maintained until $t = 15.6\ \mu$s. In Fig. S3, we present a representative timing diagram of the driving signals for the EO switches, corresponding to the case where Alice 1 and Alice 2 both measure the target qubit $|0\rangle$ in the computational basis and subsequently reprepare it in the state $|0\rangle$. In this diagram, all driving signals are initially set to 1.7 V and are reset to 1.7 V after $t = 19\ \mu$s. The rising time of all EO switches is less than 100 ns. The pulses indicate the arrival times of the photon at the corresponding EO switches: the pink pulses correspond to the order from Alice 1 to Alice 2, while the green pulses correspond to the order from Alice 2 to Alice 1.

The above timing analysis specifies the coordination required for the experimental operations. We now discuss the causal relations implied by this configuration. In the present experiment, true spacelike separation between Bob and the other three agents is not achieved, due to the lack of experimental equipment capable of performing rapid polarization measurements (*55, 56*). With such equipment, the current experimental configuration could be directly extended to realize genuine spacelike separation. Potential challenges in such an implementation may include phase disturbances, polarization fluctuations, and precise time synchronization among the devices, which are required to coordinate the operations of the agents in a field experiment.

5 The no-signaling restriction induced by the causal structure of our proposal.

The DRF inequality in Ref. (*53*) is built in the scenario with four agents involved, where two are embedded in a quantum switch Alice 1 and Alice 2, and the other two are space-like separated (Bob) or in the causal future (Charlie) from the quantum switch. In addition to the violation of the DRF inequality, experimental evidence of indefinite causal order requires that the correlations observed in the experiment obey some no-signaling conditions induced by the causal structure of the four agents. The no-signaling restriction requires that the mean value of the outcomes of an observer

remain constant when other observers change their measurement settings. The requirement of the overall causal structure on the no-signaling condition can be expressed as

$$\mathcal{NS} := \{p \in \mathcal{P}_{\vec{a}bc|\vec{x}yz} : \vec{a}c \perp\!\!\!\perp_p y \;\&\; b \perp\!\!\!\perp_p \vec{x}z\}\,, \tag{S1}$$

Where $\mathcal{P}_{\vec{a}bc|\vec{x}yz}$ is the set of conditional probability distributions and $\perp\!\!\!\perp$ denotes statistical independence. For the DRF inequality we tested (Eq. (1) of the main text), the first term of it considers the situation where Bob projects his shared photon onto the eigenstate $|H\rangle$ of the Z basis ($y = 0$, $b = 0$). This reduces the switch to a wiring in which Alice 1 is before Alice 2. The no-signaling condition for this term is

$$\mathcal{DRF}_1 := \{p \in \mathcal{NS} : a_1 b \perp\!\!\!\perp_p x_2 \;\&\; \vec{a}b \perp\!\!\!\perp_p z\}\,. \tag{S2}$$

Analogously, the second term indicates a wiring from Alice 2 to Alice 1 and the no-signaling condition is

$$\mathcal{DRF}_2 := \{p \in \mathcal{NS} : a_2 b \perp\!\!\!\perp_p x_1 \;\&\; \vec{a}b \perp\!\!\!\perp_p z\}\,. \tag{S3}$$

6 Extending to test other DRF inequalities.

In addition to the VBC inequality tested in our experiment, Ref. (*53*) also introduced several other DRF inequalities. Some of these can be (weakly) violated by the quantum switch, while others cannot. All of these inequalities are valid and tight for the DRF polytope, which characterizes correlations compatible with a hidden-variable model satisfying 'Definite Causal Order', 'Relativistic Causality', and 'Free Interventions'. These inequalities define faces—though not necessarily facets—of a lower-dimensional projection of the full DRF polytope. Our experimental setup can be naturally extended to test any of the inequalities that are violated by the quantum switch. This is because the correlations involved in these inequalities are fully determined by the joint probabilities $P(a_1, a_2, b, c|x_1, x_2, y, z)$, all of which are accessible in our setup. The only modification required is the implementation of different single-qubit measurements on the control qubit, depending on the specific inequality being tested.
